# Supplementary material for: ProteinLens: a web-based application for the analysis of allosteric signalling on atomistic graphs of biomolecules
Source: Nucleic Acids Res. 2021 May 12;49(W1):W551–8. doi: 10.1093/nar/gkab350 (PMC8661402; doi:10.1093/nar/gkab350)
Supplement: gkab350_Supplemental_Files [file gkab350_supplemental_files.zip › proteinlens_manuscript.pdf]

Published online xx Month 2021 *Nucleic Acids Research, 2021, Vol. xx, No. xx* **1–7**
doi:10.1093/nar/gkn000

ProteinLens: a web-based application for the analysis of allosteric signalling on atomistic graphs of biomolecules.

Sophia F. Mersmann [1,*], Léonie Strömich [2, *], Florian J. Song[2], Nan Wu[2], Francesca Vianello[2], Mauricio Barahona[1] and Sophia N. Yaliraki [2†]

[1]Department of Mathematics, Imperial College London, Huxley Building, 180 Queen's Gate, London SW7 2AZ, United Kingdom and [2]Department of Chemistry, Imperial College London, Molecular Science Research Hub, 82 Wood Lane, London W12 0BZ, United Kingdom

Received xx, 2021; Revised xx, 2021; Accepted xx, 2021

ABSTRACT

The investigation of allosteric effects in biomolecular structures is of great current interest in diverse areas, from fundamental biological enquiry to drug discovery. Here we present ProteinLens, a user-friendly and interactive web application for the investigation of allosteric signalling based on atomistic graph-theoretical methods. Starting from the PDB file of a biomolecule (or a biomolecular complex) ProteinLens obtains an atomistic, energy-weighted graph description of the structure of the biomolecule, and subsequently provides a systematic analysis of allosteric signalling and communication across the structure using two computationally efficient methods: Markov Transients and Bond-to-Bond propensities. ProteinLens scores and ranks every bond and residue according to the speed and magnitude of the propagation of fluctuations emanating from any site of choice (e.g., the active site). The results are presented through statistical quantile scores visualised with interactive plots and adjustable 3D structure viewers, which can also be downloaded. ProteinLens thus allows the investigation of signalling in biomolecular structures of interest to aid the detection of allosteric sites and pathways. ProteinLens is implemented in Python/SQL and freely available to use at: www.proteinlens.io.

INTRODUCTION

Allostery describes the effect of a distant binding event towards the orthosteric site activity of a protein (or protein complex) and the resulting regulation of function (1). The study of allostery is of particular interest in the context of drug discovery, where efforts targeting allosteric modes of action have gained traction over the past decades. Indeed, allosteric binding sites provide selectivity and sensitivity advantages, especially in large protein families, since allosteric sites are often less conserved within protein families and hence allow more selective targeting of disease-causing proteins (2). Additionally, allosteric modulation can both up- and down-regulate a given protein function, leading to the potential development of highly specific drugs (as reviewed by Wenthur *et al.* (3)).

However, the experimental discovery of allosterically regulated proteins is often a product of chance or requires high-throughput screenings (4). Computational methodologies that can provide a more targeted approach to investigate allosteric effects in protein structures, allowing for higher precision and for the exploration of a much wider ligand space, are of great interest for allosteric discovery. Lu *et al.* recently reviewed the available tools and databases for allosteric site detection and prediction (5). Many such methods are of little use to researchers in wet labs as they are not easily accessible, and only some of the presented methods offer easy-to-use web interfaces (6, 7, 8, 9, 10, 11, 12, 13, 14, 15).These webservers can be broadly classed into two types: they either focus on identifying allosteric sites (6, 9, 10, 11, 13, 15) or on investigating allosteric signalling and functional residues (7, 8, 12, 14, 15). The underlying methodologies for allosteric site prediction range from structure-based (9, 15), normal model analysis (10), molecular dynamics simulations (6) to correlation analysis (13) and Monte Carlo simulations (11). For the detection of allosteric signalling and mutations, the web applications are based on Monte Carlo path simulations (7), normal mode

*These authors contributed equally.
†To whom correspondence should be addressed. Tel: +44 207 5945899; Email: s.yaliraki@imperial.ac.uk

© 2021 The Author(s)
This is an Open Access article distributed under the terms of the Creative Commons Attribution Non-Commercial License (http://creativecommons.org/licenses/by-nc/2.0/uk/) which permits unrestricted non-commercial use, distribution, and reproduction in any medium, provided the original work is properly cited.

analysis (8, 14) or elastic network models (12). Most of these underlying methodologies are computationally heavy, thus impacting the user experience, since results might only be available after a considerable waiting time. In other cases, the computational cost is reduced through coarse-graining and reducing the level of detail of the interactions and structure, at the cost of detailed chemical information.

ProteinLens is a computational tool for the study of detailed signalling across atomistic structures of biomolecules such as proteins or DNA. A major application is in the study of allostery, which is often considered to be facilitated by communication within a protein (or protein complex) based on the idea that an input on a distant site on the protein, i.e. on the allosteric site, is transmitted towards the active site over intrinsic pathways (16).

ProteinLens provides a computationally inexpensive and efficient web application that addresses both aspects of allosteric signalling: for a given input site, it scores allosteric hotspots and communication pathways within the protein. The methodology underlying ProteinLens achieves atomistic resolution, which allows the identification of single important bonds and atoms. This level of detail is achieved by building atomistic graph representations of experimentally resolved biomolecular structures using BagPype (17). BagPype obtains an atomistic graph from the 3D coordinates of a protein (or a protein complex, or a protein/DNA complex) by first detecting and then weighting the graph edges according to covalent and weak interactions (hydrogen bonds, electrostatic and hydrophobic interactions) between atoms. Hence the structure is transformed into a weighted graph, where every atom is a node and all bonds and interactions are summarised as edges, thus capturing the physico-chemical properties of biological entities in atomistic detail (18, 19). These graphs are then used to compute two separate graph-theoretical measures to model connectivity and signal distribution in proteins:

- **Bond-to-bond propensity** is an edge-based measure that quantifies the magnitude of the redistribution of fluctuations between a chosen source site and every other bond in the structure. This measure has been shown to detect allosteric sites in proteins (20, 21).
- **Markov transient** is a node-based measure that models how the perturbations propagate over the atomistic graph using a random walk formalism. This allows us to capture dynamical aspects of signal propagation within the same static protein structure that serve as a means to detect allosteric signalling. Calculating the Markov transient half-times quantifies the communication between a source site and any node in the graph. This analysis has previously been used to investigate allosteric communication pathways in caspase-1 (19) and contributed to the discovery of an allosteric binding site to inhibit p90 ribosomal s6 kinase 4 (RSK4) (22).

Both methods are quantified with statistical scores computed with quantile regression.

ProteinLens presents interactive visualisations accompanied by color-coded 3D-rendered protein structures to allow the user to explore the results intuitively. To support the user in gaining problem-specific insights from the calculations, ProteinLens provides complementary entry points for data exploration, i.e., the user can choose to: (i) focus on analysis of prevalent hotspots and coldspots; (ii) inspect the most relevant residues; or (iii) visually follow the random walker, which can aid in detecting pathways. ProteinLens also provides the user with the option to score a site of interest in a comparative manner, using a bootstrapping method against randomised sites to calculate a significance level. ProteinLens thus presents a broad tool to study atomistic communication pathways and connectivity within proteins and protein multimers. The underlying theoretical methods are computationally inexpensive because of their reliance on sparse matrices, thus allowing the study of large atomistic structures. This makes them well suited to be deployed within an interactive web application.

METHODS

For a detailed description of the methodologies underpinning ProteinLens, including mathematical formulas and computational methods, see Refs. (17, 18, 19, 20, 23, 24) and the background section of ProteinLens: https://proteinlens.io/webserver/background. The following is a short overview of the theoretical background and the extended benchmarking and scoring of our methodologies.

Atomistic graph construction

ProteinLens uses BagPype (17) to construct atomistic graphs from three-dimensional coordinate data of biomolecules, as stored in the Protein Data Bank (PDB) (25). BagPype builds on previous work by Delmotte *et al.* and Amor *et al.* who studied the translation of protein structures into atomistic graphs: every atom in the structure becomes a node in the graph and all chemical bonds and interactions between these atoms are recorded as edges in the graph (18, 19). This includes the bonds and interactions that are formed between the ligand molecule and the protein. BagPype detects the following bond and interaction types: covalent bonds, hydrogen bonds, hydrophobic interactions, salt bridges, electrostatic interactions and $\pi-\pi$ stacking (in structures that contain DNA). Every edge is then weighted according to its type and strength. This approach allows us to describe physicochemical characteristics of a structure in atomistic detail.

Bond-to-bond propensity

To investigate the connectivity within a structure we calculate a measure called Bond-to-bond propensity, which quantifies the connectivity between the bonds in a user-defined source and any other bond in the structure. Bond-to-bond propensities assess the propagation of a perturbation originating at the source site through a edge formulation of random walks. For the underlying mathematical derivations, see Amor *et al.* (20). Bond-to-bond propensity is edge-based, and corresponding residue propensities are obtained by summing over the bonds in the residue. Bond-to-bond propensities have been shown to give insight into protein allostery and into cooperativity of multimeric proteins (20, 21).

Markov transient times

Another methodology accessible through ProteinLens is the computation of Markov transient times. The intuition behind this approach is to follow the path of a random walker on a protein graph, originating on the nodes contained within a chosen source. At each time step the walker will move from one node to another depending on the strength of the edge between those two nodes. We directly visualise this signalling process by calculating the probability of the random walker being at a certain node at any time point. A measure for signal propagation between the source and any node in the graph is the *characteristic transient time* $t_{1/2}$ of a node, defined as the time needed for the probability to reach half its stationary value at that node. Hence, this provides a measure of how fast the signal reaches any atom in the structure and thus of how connected such atom is to the source. This methodology has been used successfully to reveal allosteric pathways in caspase-1 (19) and aided in allosteric site discovery in RSK4 (22).

Quantile Regression

The distribution of Bond-to-bond propensities decline with distance from the chosen source, whereas Markov Transient half-times increase with distance. Whilst the former is a natural consequence of investigating diffusion processes on graphs, the latter is a result of Markov transients being based on a Markovian random walk.

To account for this distance bias, we use quantile regression into our workflow (26). This method assigns a quantile score (from 0 to 1) to every bond or atom based on the significance of that atom or bond compared to all other atoms and bonds equidistant from the source.

This allows the identification of atoms, bonds and residues that have a statistically significantly higher propensity (or lower half-time) discounting the effect of the distance from the source. This step is especially important for long-range effects like allostery. The computed quantile scores allow the comparison of residues in a structure for different settings i.e. two calculations which are sourced from different sites. More details can be found in Amor *et al.* (20) and in Ref. (23).

Benchmarking and Scoring

The methods have been benchmarked against available datasets through a range of statistical scoring measures. Note that our methods do not impose a distance cut-off onto the detection of allosteric sites, as allosterism can be conferred over short- and long-range effects (for an alternative approach see Ref. (27)). In previous work, bond-to-bond propensity correctly predicted allosteric sites in 19 out of 20 allosteric proteins with at least one of the introduced statistical scores (20). For full details on the proteins and scoring see Supplementary Information in Amor *et al.* (20). Here, we have additionally benchmarked the method against allosteric proteins from the ASBench database (28). After extracting all the structures in ASBench, 113 proteins with 118 allosteric sites contained the full information about the orthosteric sites and correct PDB structures essential to be used with our framework. Details of the orthosteric and allosteric sites were retrieved from the ASD database (Release 4.10) (29), and the proteins and site residues used can be found in Supplementary Table 1. Using the four statistical scorings introduced in Amor *et al.* (20), 102 out of 118 allosteric sites were detected correctly according to at least one statistical scoring measure (see Supplementary Table 2). This predictive accuracy of 86% by bond-to-bond propensity outperforms other reported methods (27, 30, 31, 32) that have been benchmarked against the ASBench database.

IMPLEMENTATION

ProteinLens is a user-friendly web application that facilitates easy access to methods previously developed in our group as described above. ProteinLens builds on Django (v.3.1) (33), a Python-based framework for web development, interfaced with a SQLite database backend. The frontend relies on Bootstrap (v4.3.1, https://getbootstrap.com/docs/4.3/getting-started/introduction/). Data-driven visualizations are made with help of the D3.js library (https://d3js.org), and zoomable 3D protein structures are powered by the NGL viewer (34). Figure 1 provides a schematic overview of the usage of ProteinLens. The specific steps are explained in more detail in the following.

Input

Our methods are based on structural data, which is used to create atomistic graphs. The most comprehensive source of such data is the Protein Data Bank (PDB) (25), from which the user is allowed to choose a structure directly, using the appropriate four letter PDB identifier. The user is also given the option to upload their own structural file, as long as it is provided in the official PDB format (version 3.3).

After a structure is uploaded to ProteinLens, the user is provided with some options to consider before the atomistic graph is constructed. As some of the structures in the PDB are experimentally determined by nuclear magnetic resonance spectroscopy (NMR), several models of the same protein will be present and the user will need to select the preferred one. By default, model one is selected along with all chains present in the PDB file, but water molecules and anisotropic temperature factor (ANISOU) entries are removed. These settings can be changed using intuitive check boxes and drop-down menus (some of which are listed under advanced settings). Additionally, we allow the choice between biological assemblies described for a respective structure. ProteinLens will automatically model or remove chains to build the biological assembly chosen by the user. Altogether, the input settings are then used to create an atomistic graph as described in the Methods section.

Computation settings

The next page on the ProteinLens webserver presents the user with a feedback report on the constructed graph, and allows them to choose further computational settings. At this stage, the connectivity of the constructed graph is also checked, which allows the user to select the relevant connected graph component if necessary.

Both methods in ProteinLens require a so called "source" from which to propagate the perturbation into the structure. ProteinLens has an intuitive way to choose source residues

on a structure using drop-down menus and open text fields. It will also directly suggest hetero atoms present in the structure, since ligand binding sites are generally good source site candidates. Once the user confirms their choice of source residues, they are asked to decide which computational method they want to apply on their graph: Bond-to-bond propensity, Markov transients, or both.

Results

Once the calculations are finished running in the backend, a new page is opened to present the results. In a first pane, ProteinLens gives an overview over the finished run including the chosen structure, all graph construction settings and the source residues. We also provide the user with a personalised session ID with which they can directly access their results from the welcome page if they were to refresh their browser.

The Bond-to-bond propensity and Markov transient results are then presented in different panes to provide a comprehensive insight into the data. Every visualisation incorporates an interactive structure displayed via the NGLViewer (34), which enables the user to explore the results directly on the chosen biomolecular structure.

The Hotspot view is provided for both Bond-to-bond propensities and Markov transient analysis and colours each residue of the structure according to the calculated quantile score (Fig. 1 bottom left). The data is also plotted in a separate chart, which is fully linked to the structural view. This result representation gives an overview over the data and allows identification of highly connected hotspots in the structure.

The Relevant Residues view is also available for both methods and allows the user to investigate highly scoring residues in more detail. We provide the option to look at different residue quantiles in an interactive manner and colour the relevant residues accordingly in the data plot (Fig. 1 bottom middle). This pane also links structure and data plot interactively. **The Scoring panel** allows a detailed investigation of the Bond-to-bond propensities of given sites of interest. A score can be calculated to assess the connectivity of any additional user-defined site (relative to the source residues). Such additional sites can be provided in the same manner as the source residues were chosen. We then calculate the average residue quantile score for the chosen site and compare it to the average random score calculated over 1000 surrogate sites randomly sampled over the whole structure under the assumption of having the same residue number and diameter as the site of interest. To provide statistical significance, we calculate a 95% confidence interval using a bootstrap. This workflow is further described in Amor *et al.* (20) and quantifies the relevance of a site of interest in the scope of the whole structure.

The Random Walker is the basis of Markov transients and we give the user the opportunity to follow signal propagation within the graph in an interactive manner. We colour each node of the graph according to the probability of the random walker ending up on this node after each time step (Fig. 1 bottom right). A slider allows to explore this probability distribution over all Markov transient time steps. This pane gives the user the option to assess the progression and localisation of signalling pathways over an interactive sliding timescale.

All results data can be downloaded in one compressed folder, and each result pane is accompanied by a screenshot option to capture a figure of the shown biomolecule.

Documentation

Full documentation for ProteinLens can be found on the webpage, including an extensive background section and a step-by-step tutorial. A frequently asked questions section is also provided to facilitate troubleshooting. We additionally provide a quick link to our recent COVID-19 work for which we used ProteinLens functionality (35). For further queries please email the ProteinLens focus group here: proteinlens@imperial.ac.uk.

CASE STUDY: ALLOSTERIC REGULATION IN HUMAN GLUCOKINASE.

Glucokinase (GCK) is one of four mammalian hexokinases, enzymes that phosphorylate glucose. It is expressed in human brain, liver, pancreas and small intestines and is described as an ultra-sensitive glucose sensor that interacts with insulin to regulate glucose metabolism (36). Mutations in the glucokinase gene are associated with hyper- and hypoglycemia and certain types of diabetes (37). Crucially, Glucokinase has a distinctly different enzymatic reaction rate compared to other hexokinases, which is believed to be underpinned by allosteric mechanisms. As GCK is a monomeric protein with a single active site, the so-called mnemonical mechanism was proposed (38) and validated further when GCK was crystallised in presence and absence of substrate and allosteric modulator (39). Here, we use the structure of active GCK bound to glucose and a small molecule activator to demonstrate the capability of ProteinLens to detect an allosteric site at 20 Å from the orthosteric binding site. The protein structure is sourced directly from the PDB by providing the four letter code: 1V4S (39). Once the structure is loaded, ProteinLens opens the pane for graph construction settings. In the case of 1V4S, the default settings are kept since the structure contains the biological relevant monomer and all solvent molecules can be stripped. The graph construction takes less than 10 seconds and once finished the user is automatically forwarded to the next page and greeted by a graph summary. This summary lists the chains, residues, atoms and bonds contained within the graph and indicates whether it is fully connected (which is the case for 1V4S).

The only input required for Bond-to-bond propensity and Markov transient time analysis is a source from which to start the calculations. In the case of human glucokinase, the active site is bound by a glucose molecule which can be chosen as the source. The computational methods yield a picture of the strength and the speed of connectivity in the protein in relation to the orthosteric site ligand (the glucose), and reveals allosteric signalling. After the calculations are finished, the user is forwarded to the results page. The different visualisation options provided by ProteinLens reveal different aspects of the glucokinase allostery.

For example, the hot- and coldspot view (result pane 1A) allows to identify areas of interest in a global manner (Fig. 2A) and identify the location of potential allosteric sites. By

looking at the highest scoring residues in the relevant residues view (result pane 1B) we can further narrow down which key residues are involved in the allosteric modulation (Fig. 2B). In the case of 1V4S there are large values in the experimentally-resolved allosteric site.

To attach a score to this detection, we can use panel 1C which allows to score a site of interest. We here enter the known allosteric site residues (Val62, Arg63, Met210, Ile211, Tyr214, Tyr215, Met235, Val452, Val455) and receive an average residue quantile score. This score is compared to an average random score of 1000 sampled surrogate sites of the same size. The known allosteric site of GCK scores 0.95 which is significantly higher than an average random site score of 0.53 (0.95% CI [0.52,0.54]). Figure 3 shows the scoring panel for this scoring run (PDB ID: 1V4S (39)).

CONCLUSIONS & FUTURE DEVELOPMENT

ProteinLens is a comprehensive web tool allowing the analysis of allosteric properties in proteins and DNA. We developed this platform to provide access to computational tools for the investigation of molecular structures in a user-focused way. The user is guided through a classical analysis workflow with intuitive input options and a simple interface. We also implemented a variety of visual data representations for a straight-forward evaluation of results. These interactive visualisations allow to explore allosteric sites and pathways which can be detected with our methods. ProteinLens further provides scoring of sites of interest to assess statistical relevance of allosteric sites. ProteinLens can also be used to investigate the reverse effect of allosteric regulation (27) by using the identified allosteric hotspots as ‘source’ residues in subsequent runs. The all-encompassing functionality of ProteinLens allows to predict allosteric properties in biological structures and is accessible to the wider community.

In future versions, we would like to implement an interactive source residue choice to make the tool even more intuitive to the user. Similar in style to the output structure viewer, we want to have a 3D visualisation of the chosen structure on which residues can be selected. This will help users who are not familiar with the details of PDB files but want to investigate the allostery of a protein from a known area of interest.

Furthermore, we would like to further the investigation of the impact of structural alterations onto molecular communication. We are therefore working towards implementing *in silico* mutational scans. This application would allow the mutation of every residue in the structure into Alanine, a technique commonly applied in molecular biology to investigate the contribution of residue side chains to protein function (40). As our methods are computationally efficient, it is possible to run a full alanine scan and apply both Bond-to-bond propensities and Markov transients to every mutated structure. We would then present the user with the residues which upon mutation lead to the highest divergence from the original results. This feature will give insight into the stability of signalling pathways and connectivity within a structure. Hence, ProteinLens would acquire the capability to study protein allostery upon mutation of key residues, a major interest in the field of predicting resistance in drug design (41). This could also be of aid in predicting where allosteric drug binding would be most effective (42).

SUPPLEMENTARY DATA

Supplementary Data are available at NAR Online.

FUNDING

We acknowledge the Engineering and Physical Sciences Research Council (EPSRC) award EP/N014529/1 supporting the EPSRC Centre for Mathematics of Precision Healthcare, award EP/L015498/1 and the Wellcome Trust grant award 215360/Z/19/Z.

Conflict of interest statement.

None declared.

ACKNOWLEDGEMENTS

We would like to thank Ben Amor for his invaluable contribution to the underlying code of this project. We thank everyone who helped with testing the Beta version of ProteinLens and contributed their error messages.

Authors’ contributions.

SM set up the server, and developed it with LS. LS drafted the paper. FS, NW, FV, MB and SY contributed to data analysis, method development, server design and paper writing. All authors read and approved the final manuscript.

REFERENCES

1. Guarnera, E. and Berezovsky, I. N. (2016) Allosteric sites: Remote control in regulation of protein activity. *Current Opinion in Structural Biology,* **37**, 1–8 [PubMed:26562539] [doi:10.1016/j.sbi.2015.10.004].
2. Yang, J. S., Seo, S. W., Jang, S., Jung, G. Y., and Kim, S. (2012) Rational engineering of enzyme allosteric regulation through sequence evolution analysis. *PLoS Computational Biology,* **8**(7) [PubMed:22807670] [PubMed Central:PMC3395594] [doi:10.1371/journal.pcbi.1002612].
3. Wenthur, C. J., Gentry, P. R., Mathews, T. P., and Lindsley, C. W. (2014) Drugs for Allosteric Sites on Receptors. *Annual Review of Pharmacology and Toxicology,* **54**(1), 165–184 [PubMed:24111540] [PubMed Central:PMC4063350] [doi:10.1146/annurev-pharmtox-010611-134525].
4. Hardy, J. A. and Wells, J. A. (2004) Searching for new allosteric sites in enzymes. *Current Opinion in Structural Biology,* **14**(6), 706–715 [PubMed:15582395] [doi:10.1016/j.sbi.2004.10.009].
5. Lu, S., He, X., Ni, D., and Zhang, J. (2019) Allosteric Modulator Discovery: From Serendipity to Structure-Based Design. *Journal of Medicinal Chemistry,* **14**(62), 6405–6421 [PubMed:30817889] [doi:10.1021/acs.jmedchem.8b01749].
6. Weinkam, P., Pons, J., and Sali, A. (2012) Structure-based model of allostery predicts coupling between distant sites. *Proceedings of the National Academy of Sciences of the United States of America,* **109**(13), 4875–4880 [PubMed:22403063] [PubMed Central:PMC3324024] [doi:10.1073/pnas.1116274109].
7. Kaya, C., Armutlulu, A., Ekesan, S., and Haliloglu, T. (2013) MCPath: Monte Carlo path generation approach to predict likely allosteric pathways and functional residues.. *Nucleic acids research,* **41**(Web Server issue), 249–255 [PubMed:23742907] [PubMed Central:PMC3692092] [doi:10.1093/nar/gkt284].
8. Goncearenco, A., Mitternacht, S., Yong, T., Eisenhaber, B., Eisenhaber, F., and Berezovsky, I. N. (2013) SPACER: Server for predicting allosteric communication and effects of regulation.. *Nucleic acids research,* **41**(Web Server issue), 266–272 [PubMed:23737445] [PubMed Central:PMC3692057] [doi:10.1093/nar/gkt460].
9. Huang, W., Lu, S., Huang, Z., Liu, X., Mou, L., Luo, Y., Zhao, Y., Liu, Y., Chen, Z., Hou, T., and Zhang, J. (2013) Allosite: A method for predicting allosteric sites. *Bioinformatics,* **29**(18), 2357–2359 [PubMed:23842804] [doi:10.1093/bioinformatics/btt399].
10. Panjkovich, A. and Daura, X. (2014) PARS: A web server for the prediction of Protein Allosteric and Regulatory Sites. *Bioinformatics,* **30**(9), 1314–1315 [PubMed:24413526] [doi:10.1093/bioinformatics/btu002].
11. Clarke, D., Sethi, A., Li, S., Kumar, S., Chang, R. W., Chen, J., and Gerstein, M. (2016) Identifying Allosteric Hotspots with Dynamics: Application to Inter- and Intra-species Conservation. *Structure,* **24**(5), 826–837 [PubMed:27066750] [PubMed Central:PMC4883016] [doi:10.1016/j.str.2016.03.008].
12. Li, H., Chang, Y. Y., Lee, J. Y., Bahar, I., and Yang, L. W. (2017) DynOmics: Dynamics of structural proteome and beyond. *Nucleic Acids Research,* **45**(W1), W374–W380 [PubMed:28472330] [PubMed Central:PMC5793847] [doi:10.1093/nar/gkx385].
13. Xu, Y., Wang, S., Hu, Q., Gao, S., Ma, X., Zhang, W., Shen, Y., Chen, F., Lai, L., and Pei, J. (2018) CavityPlus: A web server for protein cavity detection with pharmacophore modelling, allosteric site identification and covalent ligand binding ability prediction. *Nucleic Acids Research,* **46**(W1), W374–W379 [PubMed:29750256] [PubMed Central:PMC6031032] [doi:10.1093/nar/gky380].
14. Tan, Z. W., Guarnera, E., Tee, W. V., and Berezovsky, I. N. (2020) AlloSigMA 2: paving the way to designing allosteric effectors and to exploring allosteric effects of mutations. *Nucleic acids research,* **48**(W1), W116–W124 [PubMed:32392302] [PubMed Central:PMC7319554] [doi:10.1093/nar/gkaa338].
15. Wang, J., Jain, A., McDonald, L. R., Gambogi, C., Lee, A. L., and Dokholyan, N. V. (2020) Mapping allosteric communications within individual proteins. *Nature Communications,* **11**(1), 3862 [PubMed:32737291] [PubMed Central:PMC7395124] [doi:10.1038/s41467-020-17618-2].
16. del Sol, A., Tsai, C.-J., Ma, B., and Nussinov, R. (2009) The origin of allosteric functional modulation: multiple pre-existing pathways.. *Structure,* **17**(8), 1042–50 [PubMed:19679084] [PubMed Central:PMC2749652] [doi:10.1016/j.str.2009.06.008].
17. Song, F., Barahona, M., and Yaliraki, S. N. (2021) `BagPype`: A Python package for the construction of atomistic, energy-weighted graphs from biomolecular structures. *figshare,* (Preprint) [doi:10.6084/m9.figshare.14039723.v1].
18. Delmotte, A., Tate, E. W., Yaliraki, S. N., and Barahona, M. (2011) Protein multi-scale organization through graph partitioning and robustness analysis: application to the myosin-myosin light chain interaction. *Physical Biology,* **8**(5), 055010 [PubMed:21832797] [doi:10.1088/1478-3975/8/5/055010].
19. Amor, B., Yaliraki, S. N., Woscholski, R., and Barahona, M. (2014) Uncovering allosteric pathways in caspase-1 using Markov transient analysis and multiscale community detection. *Molecular BioSystems,* **10**(8), 2247–2258 [PubMed:24947802] [doi:10.1039/c4mb00088a].
20. Amor, B. R. C., Schaub, M. T., Yaliraki, S. N., and Barahona, M. (2016) Prediction of allosteric sites and mediating interactions through bond-to-bond propensities. *Nature Communications,* **7**, 12477 [PubMed:27561351] [PubMed Central:PMC5007447] [doi:10.1038/ncomms12477].
21. Hodges, M., Barahona, M., and Yaliraki, S. N. (2018) Allostery and cooperativity in multimeric proteins: bond-to-bond propensities in ATCase. *Scientific Reports,* **8**(1), 11079 [PubMed:30038211] [PubMed Central:PMC6056424] [doi:10.1038/s41598-018-27992-z].
22. Chrysostomou, S., Roy, R., Prischi, F., Chapman, K., Mufti, U., Mauri, F., Bellezza, G., Abrahams, J., Ottaviani, S., Castellano, L., Giamas, G., Hrouda, D., Winkler, M., Klug, D., Yaliraki, S., Barahona, M., Wang, Y., Ali, M., Seckl, M., and Pardo, O. (2019) Abstract 1775: Targeting RSK4 prevents both chemoresistance and metastasis in lung cancer. *Cancer Research,* **79**(13 Supplement), 1775 [doi:10.1158/1538-7445.AM2019-1775].
23. Amor, B. R. C. Exploring allostery in proteins with graph theory. PhD thesis Imperial College London (2016) [doi:10.25560/58214].
24. Delmotte, A. All-scale structural analysis of biomolecules through dynamical graph partitioning. PhD thesis Imperial College London (2014) [doi:10.25560/29861].
25. Berman, H. M., Westbrook, J., Feng, Z., Gilliland, G., Bhat, T. N., Weissig, H., Shindyalov, I. N., and Bourne, P. E. (2000) The Protein Data Bank.. *Nucleic Acids Research,* **28**(1), 235–242 [PubMed:10592235] [PubMed Central:PMC102472] [doi:10.1093/nar/28.1.235].
26. Koenker, R. (2005) Quantile regression, Cambridge University Press, .
27. Tee, W.-V., Guarnera, E., and Berezovsky, I. N. (2018) Reversing allosteric communication: From detecting allosteric sites to inducing and tuning targeted allosteric response. *PLOS Computational Biology,* **14**(6), 1–26 [PubMed:29912863] [PubMed Central:PMC6023240] [doi:10.1371/journal.pcbi.1006228].
28. Huang, W., Wang, G., Shen, Q., Liu, X., Lu, S., Geng, L., Huang, Z., and Zhang, J. (2015) ASBench: benchmarking sets for allosteric discovery. *Bioinformatics,* **31**(15), 2598–2600 [PubMed:25810427] [doi:10.1093/bioinformatics/btv169].
29. Liu, X., Lu, S., Song, K., Shen, Q., Ni, D., Li, Q., He, X., Zhang, H., Wang, Q., Chen, Y., Li, X., Wu, J., Sheng, C., Chen, G., Liu, Y., Lu, X., and Zhang, J. (2019) Unraveling allosteric landscapes of allosterome with ASD. *Nucleic Acids Research,* **48**(D1), D394–D401 [PubMed:31665428] [doi:10.1093/nar/gkz958].
30. Greener, J. G. and Sternberg, M. J. (2015) AlloPred: prediction of allosteric pockets on proteins using normal mode perturbation analysis. *BMC Bioinformatics,* **16**(1), 335 [PubMed:26493317] [PubMed Central:PMC4619270] [doi:10.1186/s12859-015-0771-1].
31. Ma, X., Meng, H., and Lai, L. (2016) Motions of Allosteric and Orthosteric Ligand-Binding Sites in Proteins are Highly Correlated. *Journal of Chemical Information and Modeling,* **56**(9), 1725–1733 [PubMed:27580047] [doi:10.1021/acs.jcim.6b00039].
32. Song, K., Liu, X., Huang, W., Lu, S., Shen, Q., Zhang, L., and Zhang, J. (2017) Improved Method for the Identification and Validation of Allosteric Sites. *Journal of Chemical Information and Modeling,* **57**(9), 2358–2363 [PubMed:28825477] [doi:10.1021/acs.jcim.7b00014].
33. Django Software Foundation, Django v. 3.1. (2020).
34. Rose, A. S., Bradley, A. R., Valasatava, Y., Duarte, J. M., Prlic, A., and Rose, P. W. (2018) NGL viewer: Web-based molecular graphics for large complexes. *Bioinformatics,* **34**(21), 3755–3758 [PubMed:29850778] [PubMed Central:PMC6198858] [doi:10.1093/bioinformatics/bty419].
35. Strömich, L., Wu, N., Barahona, M., and Yaliraki, S. N. (2020) Allosteric Hotspots in the Main Protease of SARS-CoV-2. *bioRxiv,* p. 2020.11.06.369439 [doi:10.1101/2020.11.06.369439].
36. Iynedjian, P. B. (2009) Molecular physiology of mammalian glucokinase. *Cellular and Molecular Life Sciences,* **66**(1), 27–42 [PubMed:18726182]

[PubMed Central:PMC2780631] [doi:10.1007/s00018-008-8322-9].

37. Šimčíková, D., Kocková, L., Vackářová, K., Těšínský, M., and Heneberg, P. (2017) Evidence-based tailoring of bioinformatics approaches to optimize methods that predict the effects of nonsynonymous amino acid substitutions in glucokinase. *Scientific Reports,* **7**(1), 1–11 [PubMed:28842611] [PubMed Central:PMC5573313] [doi:10.1038/s41598-017-09810-0].
38. Storer, A. C. and Cornish Bowden, A. (1977) Kinetic evidence for a 'Mnemonical' mechanism for rat liver glucokinase. *Biochemical Journal,* **165**(1), 61–69 [PubMed:889576] [PubMed Central:PMC1164869] [doi:10.1042/bj1650061].
39. Kamata, K., Mitsuya, M., Nishimura, T., Eiki, J.-i., and Nagata, Y. (2004) Structural Basis for Allosteric Regulation of the Monomeric Allosteric Enzyme Human Glucokinase. *Structure,* **12**(3), 429–438 [PubMed:15016359] [doi:10.1016/j.str.2004.02.005].
40. Morrison, K. L. and Weiss, G. A. (2001) Combinatorial alanine-scanning. *Current Opinion in Chemical Biology,* **5**(3), 302–307 [PubMed:11479122] [doi:10.1016/s1367-5931(00)00206-4].
41. Brankin, A. E. and Fowler, P. W. (2019) Predicting Resistance Is (Not) Futile. *ACS Central Science,* **5**(8), 1312–1314 [PubMed:31482113] [PubMed Central:PMC6716340] [doi:10.1021/acscentsci.9b00791].
42. Guarnera, E. and Berezovsky, I. N. (2020) Allosteric drugs and mutations: chances, challenges, and necessity. *Current Opinion in Structural Biology,* **62**, 149–157 [PubMed:32062398] [doi:10.1016/j.sbi.2020.01.010].

FIGURE CAPTIONS

Figure 1

Flowchart of the ProteinLens process. 1) The user can upload their own structure file in .pdb format or source it directly from the PDB using a 4 letter identifier. ProteinLens then provides graph construction settings to adjust the biomolecular structure or strip certain atoms or residues. 2) After the graph is constructed, the user provides interactively a set of source residues and choose which methodology to run. By default both Bond-to-bond propensity and Markov transient time are chosen. 3) In the final step, the user is provided with a variety of complementary visualisations, each of which provide an insight into a different aspect of allosteric signalling. ProteinLens also provides a scoring feature to access the significance of a previously known site.

Figure 2

Hotspot and relevant residues views for human glucokinase. Showcased here are the Bond-to-bond propensity results when sourced from the active site ligand in GCK(PDB ID: 1V4S (39)). A) The hotspot view allows to find areas of high or low connectivity to the source site (green). We plot all data points as propensity over distance from source and provide a 3D protein structure. Both are coloured according to quantile score and are fully linked to highlight residues and data points in an interactive manner. B) The relevant residues view highlights the highest scoring residues with an adjustable quantile score cut off. As above, the two plots are fully linked to allow interactive access to single data points.

Figure 3

Screenshot of result pane for scoring the allosteric site of human glucokinase. This pane uses the propensity results of a given run and shown here is the GCK structure (PDB ID: 1V4S (39)) after a run sourced from the active site ligand. All residues belonging to a site of interest can be entered via a dropdown menu or an open text field. The pane lists the results for each site of interest highlighted on the structure and provides information on average quantile scores of the sites. It also provides a randomly generated site score to compare against.
