# Supplementary material for: ProteinLens: a web-based application for the analysis of allosteric signalling on atomistic graphs of biomolecules
Source: Nucleic Acids Res. 2021 May 12;49(W1):W551–8. doi: 10.1093/nar/gkab350 (PMC8661402; doi:10.1093/nar/gkab350)
Supplement: gkab350_Supplemental_Files [file gkab350_supplemental_files.zip › Supplementary_Data.pdf]

Supplementary Table 1: **Details of proteins collected from the ASD and ASBench databases.** For proteins with PDB ID 1CE8, 1Z8D, 2Q8M, 3ETE and 3KGF, there are two distinct allosteric sites reported.

Protein	PDB	Allosteric Ligands	Allosteric Ligand Number	Allosteric Site Residues	Active Site Residues
**Seminal ribonuclease**	11BG	U2G	A131	A14,A24,A27,A28,A94,A95,B32,B33	A41,A43,A44,A45,A46,A66,A81,A83,A85,A100,A102,A104,A119,A120,A121,A122,A123,A124,B12
**Pyruvate kinase 1**	1A3W	FBP	A1007	A401,A402,A403,A404,A405,A406,A407,A408,A452,A459,A483,A484,A490,A491,A492	A49,A51,A53,A84,A85,A89,A91,A213,A214,A240,A241,A242,A261,A262,A263,A264,A265,A266,A267,A297,A298,A330,A332
**Hemoglobin subunit beta**	1B86	DG2	D701	B145,B225,D545,D625	A29,A43,A58,A62,A87,A101
**Carbamoyl-phosphate synthase large chain**	1CE8_1	ORN	A5011	A783,A791,A793,A892,A893,A895,A907,A1039,A1040,A1041,A1042	A690,A713,A715,A725,A727,A753,A754,A755,A756,A757,A761,A781,A784,A785,A786,A787,A788,A789,A790,A792,A829,A831,A840,A841,A843,A848,A908,A909,A910
**Carbamoyl-phosphate synthase large chain**	1CE8_2	IMP	A5012	A948,A949,A954,A974,A975,A976,A977,A993,A994,A995,A1001,A1015,A1016,A1017,A1025,A1026,A1028,A1029,A1030	A690,A713,A715,A725,A727,A753,A754,A755,A756,A757,A761,A781,A784,A785,A786,A787,A788,A789,A790,A792,A829,A831,A840,A841,A843,A848,A908,A909,A910
**Ribose-phosphate pyrophosphokinase**	1DKU	AP2	A1002	A140,A148,A149,A310,A311,A312,A315,B105,B106,B107,B108,B109	A99,A101,A102,A103,A104,A106,A107,A110,A135,A174,A227,B138

**Glycogen phosphorylase, liver form**	1EM6	CP4	A862	A37,A38,A40,A60,A63,A64,A67,A188,A189,A190,A191,A229,B38,B40,B60,B63,B64,B67,B188,B189,B190,B191,B229	A134,A135,A675,A676
**Fructose-1,6-bisphosphatase 1**	1FRP	AMP	A338	A17,A20,A21,A24,A26,A27,A28,A29,A30,A31,A112,A113,A140,A160,A177	A121,A122,A124,A125,A212,A215,A244,A246,A247,A248,A249,A250,A251,A252,A262,A264,A269,A274,A275,A280,B241,B242,B243
**Glucose-1-phosphate thymidylyltransferase**	1G3L	TRH	A501	A45,A114,A115,A116,A117,A118,A119,A120,A250,A251,A255,A256,A259,A293,C216,C218,C219,C220	A8,A9,A10,A11,A12,A13,A14,A15,A16,A25,A26,A54,A55,A82,A84,A85,A86,A87,A88,A90,A108,A109,A110,A111,A162,A194,A196,A225,A227
**NAD-dependent malic enzyme, mitochondrial**	1GZ3	FUM	A605	A64,A67,A88,A91,A95,B127,B128	A112,A165,A166,A167,A168,A279,A419,A420,A421,A422,A466,A467
**Glucose-1-phosphate thymidylyltransferase 1**	1H5S	TMP	A1292	A46,A115,A116,A117,A118,A250,A251,A252,A256,A257,A260,D219,D220,D221	A8,A9,A10,A11,A12,A13,A14,A15,A16,A25,A26,A54,A55,A82,A84,A85,A86,A87,A88,A90,A108,A109,A110,A111,A162,A194,A196,A225,A227
**Anaerobic ribonucleoside-triphosphate reductase**	1H78	DCP	A1589	A98,A99,A100,A102,A103,A104,A107,A110,A111,A114,A146	A58,A64,A65,A66,A67,A68,A69,A70,A441,A443,A444,A445,A446,A447,A448,A449,A450,A451,A580,A581
**Anthranilate synthase component 1**	1I7S	TRP	A601	A38,A39,A40,A49,A50,A291,A292,A293,A453,A454,A455,A463,A465	A258,A259,A260,A262,B11,B56,B57,B58,B59,B60,B61,B62,B84,B85,B86,B87,B89,B107,B133,B134,B135,B136,B137,B170,B172
**Hemoglobin subunit alpha**	1IWH	PEM	A501	A57,A60,A61,A64,A65,A83	A29,A43,A58,A62,A87,A101
**Uracil**	1JLR	GTP	A303	A78,A101,A103,A104,A105,A124,A125,A12	B113,B166,B167,B168,B227,B228,B235,

**phosphoribosyltransferase**				9,A158,C44,C65,C68,D59	B236,B237
**Phospho-2-dehydro-3-deoxyheptonate aldolase, Phe-sensitive**	1KFL	PHE	A1354	A150,A151,A154,A178,A179,A180,A209,A211,A214,A221,B6,B7,B10	A61,A92,A94,A96,A97,A98,A143,A161,A162,A163,A164,A186,A189,A234,A265,A267,A268,A269,A302,A326
**Glucose-1-phosphate thymidylyltransferase**	1LVW	TYD	A3002	A43,A112,A113,A114,A115,A116,A117,A247,A248,A249,A253,A254,C217	A8,A9,A10,A11,A12,A13,A14,A15,A16,A25,A26,A54,A55,A82,A84,A85,A86,A87,A88,A90,A108,A109,A110,A111,A162,A194,A196,A225,A227
**Sulfate adenylyltransferase**	1M8P	PPS	A574	A405,A434,A437,A446,A451,A454,A455,A476,A477,A478,A479,A515,A517,A526,A527,A528,A529,A530	A196,A197,A198,A199,A200,A205,A206,A209,A265,A267,A276,A289,A290,A291,A292,A293,A294,A295,A296,A330,A331,A332,A333,A334
**Glucose-1-phosphate thymidylyltransferase**	1MP3	TTP	A501	A46,A115,A116,A117,A118,A119,A120,A252,A256,A257,A260,B219,B220,B221	A8,A9,A10,A11,A12,A13,A14,A15,A16,A25,A26,A54,A55,A82,A84,A85,A86,A87,A88,A90,A108,A109,A110,A111,A162,A194,A196,A225,A227
**Glucosamine-6-phosphate isomerase 1**	1NE7	16G	B2299	A1,A2,A258,A262,B151,B152,B158,B159,B160,B161,B184	A39,A40,A41,A42,A43,A44,A45,A71,A72,A85,A136,A137,A138,A139,A140,A143,A144,A145,A146,A166,A170,A172,A173,A207,A208
**ATP phosphoribosyltransferase**	1NH8	HIS	A289	A216,A217,A218,A242,A273,A275	A11,A12,A70,A71,A88,A89,A90,A116,A155,A156,A157,A158,A159,A160,A161,A162
**Ornithine decarboxylase**	1NJJ	GET	A601	A22,A243,A339,A340,A341,A382,A384,A385	A67,A69,A70,A88,A111,A113,A154,A197,A199,A200,A235,A236,A237,A238,A274,A275,A276,A277,A278,A331,A332,A

					333,A389,B323,B360,B361,B362,B363
**Phospho-2-dehydro-3-deoxyheptonate aldolase, tyrosine-inhibited**	1OF6	DTY	A1370	A162,A166,A169,A193,A194,A195,A224,A226,A227,B21,B22,B25,B28	A61,A92,A94,A96,A97,A98,A143,A161,A162,A163,A164,A186,A189,A234,A265,A267,A268,A269,A302,A326
**ATP-dependent 6-phosphofructokinase isozyme 1**	1PFK	ADP	A326	A154,A158,A185,A187,A211,A212,A213,A214,A215,A319,B21,B25,B54,B55,B58,B59	A9,A10,A11,A12,A41,A71,A72,A73,A74,A75,A76,A77,A82,A101,A102,A103,A104,A105,A106,A107,A108,A109,A111,A124,A125,A129,A171
**Parathion hydrolase**	1QW7	EBP	A702	A51,A350	A55,A57,A131,A132,A201,A202,A230,A254,A301
**Tyrosine-protein phosphatase non-receptor type 1**	1T49	892	A301	A189,A192,A193,A196,A197,A200,A276,A277,A279,A280,A281,A282	A45,A46,A47,A48,A49,A111,A115,A120,A180,A181,A182,A215,A216,A217,A218,A219,A220,A221,A222,A262,A266
**NAD(P)-dependent glyceraldehyde-3-phosphate dehydrogenase**	1UXV	AMP	A1503	A72,A79,A132,A133,A134,A135,A154,A155,A156,A157,A184,A479	A166,A168,A242,A296,A297,A397
**Cytochrome P450 3A4**	1W0F	STR	A1499	A213,A214,A217,A219,A220,A240	A94,A105,A118,A119,A120,A126,A130,A137,A184,A271,A301,A302,A303,A305,A306,A307,A309,A310,A311,A313,A364,A368,A369,A370,A372,A373,A374,A375,A433,A434,A435,A436,A437,A439,A440,A441,A442,A443,A444,A445,A447,A448,A452
**Response regulator PleD**	1W25	C2E	A503,A505	A148,A153,A174,A175,A177,A178,A356,A357,A358,A359,A360,A362,A377,A379,A383,A387,A390	A294,A331,A332,A335,A339,A340,A341,A342,A343,A344,A347,A366,A368,A369,A370,A371

**Acetyl-CoA carboxylase**	1W96	S1A	A1567	A69,A73,A76,A77,A389,A392,A393,A396,A397,A398,A454,A485,A487,A510,A512	A189,A352,A363,A364,A365,A366,A377,A378,A379,A380,A381
**Glycogen phosphorylase, muscle form**	1Z8D_1	AMP	A900	A67,A71,A75,A309,A310,A315,A316,A317,A318	A134,A135,A675,A676
**Glycogen phosphorylase, muscle form**	1Z8D_2	ADE	A902	A282,A285,A610,A612,A613	A134,A135,A675,A676
**Copper-containing nitrite reductase**	1ZDS	ACM	A2500	A47,A60,A62,A93,A95,A144,A145,A148,A199	A98,A100,A106,A135,A137,A142
**4-hydroxy-tetrahydrodipicolinate synthase**	2ATS	DLY	A3003	B83	A8,A40,A43,A44,A45,A46,A101,A133,A161,A186,A203,A204,A205,A248
**Aspartate carbamoyltransferase regulatory chain**	2BE9	CTP	B401	B14,B15,B20,B22,B23,B64,B88,B90,B93,B95,B98	A48,A50,A51,A52,A53,A54,A55,A56,A105,A127,A130,A134,A137,A167,A168,A228,A229,A230,A231,A233,A234,A265,A266,A267,A268,A296
**Pyruvate dehydrogenase kinase isozyme 2**	2BU8	TF4	A1379	A53,A80,A111,A112,A115,A154,A157,A158,A161	A243,A244,A246,A247,A248,A250,A251,A282,A284,A285,A286,A287,A295,A301,A315,A316,A317,A318,A319,A320,A321,A322,A323,A338,A340,A346
**Serum albumin**	2BXA	C1F	A2001	A150,A199,A214,A218,A219,A222,A223,A238,A242,A257,A260,A264,A290,A291	A387,A388,A391,A392,A395,A403,A407,A410,A411,A414,A430,A431,A433,A434,A435,A437,A438,A449,A450,A453,A457,A485,A488,A489
**Hemoglobin subunit beta**	2D60	L35	B1200	A36,A99,A100,A103,B35,B37,B108,C95,C137,C141	A29,A43,A58,A62,A87,A101
**L-asparaginase 1**	2HIM	ASN	A8001	A162,A240,A271,A272,A273,A301,A302,A3	A12,A13,A14,A17,A58,A59,A60,A61,A6

				03,C240	2,A89,A90,A91,A92,A93,A163,C246,C247
**Putative deoxycytidylate deaminase**	2HVW	DCP	A1201	A21,A23,A43,A44,A46,A47,A49,A50,A53,A75,B108	A24,A26,A27,A28,A29,A44,A45,A46,A54,A61,A64,A65,A66,A67,A69,A70,A71,A72,A73,A94,A96,A97,A98,A99,A102,A120,A121
**Glycogen phosphorylase, muscle form**	2IEG	FRY	A901	A60,A63,A67,A188,A190,A191,A192,A194,A229,B37,B38,B39,B40,B53,B57,B185,B186,B188	A134,A135,A675,A676
**Lysine-sensitive aspartokinase 3**	2J0X	LYS	A1451	A318,A321,A323,A324,A325,A344,A345,A346,B338,B339,B340	A8,A9,A10,A11,A12,A13,A39,A199,A202,A219,A220,A221,A222,A223,A225,A226,A227,A228,A229,A230,A231,A232,A251,A255,A256,A257,A258,A259,A301,A302
**Myosin-2 heavy chain**	2JHR	PBQ	A1780	A265,A420,A423,A424,A427,A428,A431,A590,A592,A617,A618,A619,A620	A179,A180,A181,A182,A183,A185,A186,A227,A233,A235,A236,A237,A454,A455,A457
**Tryptophan 2,3-dioxygenase**	2NW8	TRP	A308	A85,A92,A220,A221,A224,A225,A228	A51,A55,A113,A117,A123,A124,A125,A248,A252,A253,A254,A255,B24
**D-3-phosphoglycerate dehydrogenase**	2PA3	SER	A451	A344,A346,A347,A348,A349,A350,A351,A370	A84,A105,A106,A108,A109,A112,A157,A158,A159,A160,A161,A162,A163,A180,A181,A182,A183,A185,A209,A210,A211,A212,A213,A214,A216,A217,A220,A238,A239,A240,A264,A265,A292,A293,A294,A295,A296
**Glutamine--fructose-6-**	2PUV	UD1	B5003	B372,B383,B384,B474,B476,B479,B484,B48	A403,A404,A405,A406,A449,A450,A451

**phosphate aminotransferase**				7,B488,B489,B490,B491,B492	,A452,A453,A454,A455,A458,A483,A501,A502,A503,A510,A587,A588,A591,B604,B605
**Indole-3-pyruvate decarboxylase**	2Q5O	PPY	A5003	A60,A214,A215,A238,A240,A241,A242,A375,A394,A395,A396,A397	A380,A401,A402,A403,A461,A462,B23,B24,B25,B71,B74,B113
**Fructose-1,6-bisphosphatase class 1**	2Q8M_1	BG6	A340	A207,A210,A221,A222,A225	A121,A122,A124,A125,A212,A215,A244,A246,A247,A248,A249,A250,A251,A252,A262,A264,A269,A274,A275,A280,B241,B242,B243
**Fructose-1,6-bisphosphatase class 1**	2Q8M_2	AMP	A341	A8,A11,A12,A15,A18,A19,A20,A21,A22,A23,A104,A105,A132,A171	A121,A122,A124,A125,A212,A215,A244,A246,A247,A248,A249,A250,A251,A252,A262,A264,A269,A274,A275,A280,B241,B242,B243
**Prephenate dehydratase**	2QMX	PHE	A303	A224,A225,A226,A227,A228,B206,B207,B209,B210,B211,B230,B240,B242	A52,A53,A54,A55,A56,A57,A79,A80,A81,A82,A83,A166,A167,A168,A169,A170
**Androgen receptor**	2QPY	4HY	A1	A723,A724,A727,A826,A829,A830,A833,A834,A837,A840	A701,A704,A705,A706,A707,A708,A711,A741,A742,A745,A746,A749,A752,A763,A764,A765,A780,A787,A873,A876,A877,A880,A891,A895,A899
**Ribonucleoside-diphosphate reductase 1 subunit alpha**	2R1R	TTP	A762	A232,A233,A234,A262,A268,A269,A275,A276,B249	A155,A207,A208,A209,A210,A224,A225,A226,A251,A252,A253,A254,A301,A437,A438,A439,A441,A442,A464,A620,A621,A622,A623,A624,A625,A626,A694
**Acetylglutamate kinase, chloroplastic**	2RD5	ARG	A1000	A33,A210,A232,A284,A285,A287,A288,A289,A290,A291,A292,A294	A41,A74,A75,A76,A77,A80,A81,A93,A96,A97,A98,A100,A108,A112,A156,A181,A192,A193,A194,A195,A196

**Uridylate kinase**	2V4Y	GTP	A1242	A92,A93,A96,A101,A102,A103,A130,E119,E120,E123,E124,E127,F72,F75	A15,A16,A17,A18,A19,A55,A56,A57,A58,A59,A61,A62,A63,A64,A73,A76,A77,A78,A80,A84,A136,A137,A138,A139,A140,A141,A142,A143,A144,A145,A146,A147,A148,A163,A201
**ATP phosphoribosyltransferase**	2VD3	HIS	A1290	A235,A236,A237,A238,A240,A254,A255,A256	A11,A12,A70,A71,A88,A89,A90,A116,A155,A156,A157,A158,A159,A160,A161,A162
**Pyruvate kinase PKLR**	2VGI	FBP	A1574	A474,A475,A476,A477,A478,A479,A480,A525,A532,A557,A559,A560,A561,A562,A563,A564,A565	A116,A118,A120,A156,A157,A161,A286,A287,A313,A315,A334,A335,A336,A337,A338,A339,A340,A370,A371,A403,A405
**Glutamate racemase**	2VVT	I24	A1269	A14,A15,A38,A152,A153,A155,A157,A187,A190,A246,A250,A254	A7,A8,A9,A11,A12,A37,A38,A39,A40,A41,A69,A70,A71,A72,A73,A116,A119,A146,A150,A180,A181,A182,A183
**Glutamate racemase**	2W4I	VGA	B1256	A38,A41,A143,A146,A147,A150,A151,B37,B38,B41,B117,B146,B147,B150,B151	A7,A8,A9,A11,A12,A37,A38,A39,A40,A41,A69,A70,A71,A72,A73,A116,A119,A146,A150,A180,A181,A182,A183
**Cytosolic purine 5'-nucleotidase**	2XJC	B4P	A1490	A144,A145,A152,A154,A354,A358,A362,A453,A456,A457	A52,A53,A54,A55,A56,A65,A151,A155,A157,A158,A161,A202,A205,A206,A207,A209,A210,A214,A215,A248,A249,A250,A251,A252,A255,A292,A348,A351,A356
**Multifunctional 2-oxoglutarate metabolism enzyme**	2Y0P	ACO	A2228	A822,A827,A830,A1035,A1037,A1038,A1054,A1058,A1060,A1062,A1092,A1142,A1145,A1146,A1147,A1148,A1149,A1150	A504,A506,A539,A540,A576,A578,A579,A602,A603,A604,A605,A606,A607,A643,A644,A645,A646,A647,A648,A651,A6

					76,A678,A680,A681,A682,A743,A747,B901,B902,B950,B952,B976,B977,B980,B1019,B1020
**Androgen receptor**	2YHD	AV6	A1921	A716,A720,A730,A733,A734,A738,A894,A898	A701,A704,A705,A706,A707,A708,A711,A741,A742,A745,A746,A749,A752,A763,A764,A765,A780,A787,A873,A876,A877,A880,A891,A895,A899
**Androgen receptor**	2YLO	YLO	A1922	A723,A724,A725,A726,A727,A826,A829,A830,A833,A834	A701,A704,A705,A706,A707,A708,A711,A741,A742,A745,A746,A749,A752,A763,A764,A765,A780,A787,A873,A876,A877,A880,A891,A895,A899
**Glycogen phosphorylase, muscle form**	3BCR	AZZ	A940	A282,A285,A610,A612,A613	A134,A135,A675,A676
**Myosin-2 heavy chain**	3BZ7	BL4	A800	A238,A239,A240,A261,A262,A263,A264,A455,A456,A467,A470,A471,A474,A634,A637,A638	A179,A180,A181,A182,A183,A185,A186,A227,A233,A235,A236,A237,A454,A455,A457
**Probable aspartokinase**	3C1N	THR	A471	A414,A416,A417,A419,A420,A421,A440,A444,B434,B435	A6,A7,A8,A9,A10,A11,A40,A41,A43,A208,A210,A211,A228,A229,A230,A231,A232,A234,A235,A236,A237,A238,A239,A240,A241,A260,A264,A265,A266,A267,A268,A311,A312,A313
**Glycogen phosphorylase, liver form**	3CEH	AVE	A833	A67,A68,A71,A72,A75,A191,A193,A227,B39,B40,B41,B42,B44,B45	A134,A135,A675,A676
**Chorismate mutase**	3CSM	TRP	A300	A71,A74,A75,A76,A79,A82,A98,A100	A12,A16,A19,A157,A164,A168,A192,A193,A194,A195,A197,A198,A201,A234,A238,A239,A240,A242,A243,A246

**Amino-acid acetyltransferase**	3D2P	ARG	A438	A17,A201,A220,A221,A257,A270,A271,A272,A273,A274,A275,A276,A277,A278,A279,A280,A334	A307,A312,A354,A355,A356,A357,A358,A359,A363,A364,A365,A366,A367,A368,A369,A370,A371,A391,A392,A394,A395,A396,A397,A398,A399,A401,A402
**D-3-phosphoglycerate dehydrogenase**	3DC2	SER	A600	A461,A463,A464,A465,A466,A467,A468,A487	A84,A105,A106,A108,A109,A112,A157,A158,A159,A160,A161,A162,A163,A180,A181,A182,A183,A185,A209,A210,A211,A212,A213,A214,A216,A217,A220,A238,A239,A240,A264,A265,A292,A293,A294,A295,A296
**Glycogen phosphorylase, muscle form**	3E3N	AMP	A843	A67,A71,A75,A309,A310,A315,A316,A317,A318,B42,B44,B45	A134,A135,A675,A676
**Uridylate kinase**	3EK5	GTP	E2006	D115,D117,D118,D121,D125,E98,E100,E108,E109,E110,E111,E112,E118,E125,E127	A15,A16,A17,A18,A19,A55,A56,A57,A58,A59,A61,A62,A63,A64,A73,A76,A77,A78,A80,A84,A136,A137,A138,A139,A140,A141,A142,A143,A144,A145,A146,A147,A148,A163,A201
**Isocitrate dehydrogenase kinase/phosphatase**	3EPS	AMP	A1604	A101,A104,A105,A113,A116,A291,A294,A295,A298,A375,A376,A377,A378	A315,A316,A317,A318,A319,A320,A321,A322,A323,A324,A325,A334,A336,A346,A348,A353,A357,A416,A417,A418,A419,A420,A421,A423,A424,A457,A461,A462,A474,A475,A476,A477,A478
**Glutamate dehydrogenase 1, mitochondrial**	3ETE_1	GTP	A553	A186,A187,A190,E150,E154,E186,E187,E189,E190	A111,A114,A126,A167,A168,A211,A349,A374,A377,A378
**Glutamate dehydrogenase 1, mitochondrial**	3ETE_2	H3P	A552,B552,C552,C5	A212,A213,A217,A257,A258,A261,A262,A265,A292,A446,A450	A111,A114,A126,A167,A168,A211,A349,A374,A377,A378

			54,D552,F552		
**Glutamate dehydrogenase 1, mitochondrial**	3ETG	GWD	A552,E552	A142,A146,A147,A150,A181,A185	A111,A114,A126,A167,A168,A211,A349,A374,A377,A378
**Leukotriene A-4 hydrolase**	3FUD	692	A710	A24,A25,A26,A35,A36,A161,A180,A182,A188	A134,A136,A137,A266,A267,A268,A269,A270,A271,A291,A292,A293,A295,A296,A299,A314,A318,A321,A322,A325,A375,A378,A383,A563,A565
**Casein kinase II subunit alpha**	3H30	RFZ	A337	A39,A40,A41,A67,A69,A101,A103,A104,A110	A45,A46,A47,A48,A51,A53,A66,A68,A95,A113,A116,A117,A118,A119,A120,A123,A160,A163,A174,A175
**Pyruvate kinase PKM**	3H6O	FBP	A541	A431,A432,A433,A434,A436,A437,A482,A489,A514,A516,A517,A518,A519,A520,A521,A522	A73,A75,A77,A113,A114,A118,A243,A270,A272,A291,A292,A293,A294,A295,A296,A297,A327,A328,A360,A362
**Glutamate racemase**	3HFR	6JZ	A270	A153,A155,A156,A246,A250	A7,A8,A9,A11,A12,A37,A38,A39,A40,A41,A69,A70,A71,A72,A73,A116,A119,A146,A150,A180,A181,A182,A183
**Toxin A**	3HO6	IHP	A270	A35,A37,A57,A60,A61,A105,A107,A154,A211,A212,A224,A235,A252,A253	A44,A46,A47,A49,A50,A108,A109,A110,A111,A112,A120,A153,A154,A155,A156,A199,A200,A201,A202,A203,A204,A205,A218
**Pyruvate kinase**	3HQP	FDP	A700	A399,A400,A401,A402,A404,A405,A453,A456,A480,A481,A485,A486,A487,A488,A489	A26,A27,A28,A29,A49,A50,A51,A53,A54,A55,A59,A60,A83,A84,A88,A90,A144,A145,A172,A173,A174,A175,A176,A211,A212,A238,A240,A264,A296,A330,A331,A332,A334,A335

**Fructose-1,6-bisphosphatase isozyme 2**	3IFA	AMP	A339	A17,A20,A21,A24,A26,A27,A28,A29,A30,A31,A112,A113,A140,A177	A121,A122,A124,A125,A212,A215,A244,A246,A247,A248,A249,A250,A251,A252,A262,A264,A269,A274,A275,A280,B241,B242,B243
**HD domain protein**	3IRH	DGT	A458	A54,A55,A247,A326,A330,A422,B14,B15,B16,B35,B36,B41,B44,B64	B48,B49,B50,B51,B52,B63,B66,B111,B114,B118,B119,B122,B129,B183,B184,B187,B191,B235,B239,B242,B243,B244,B248,B252,B368,B369
**Tyrosine-protein kinase ABL1**	3K5V	STJ	A1	A356,A359,A360,A363,A448,A451,A452,A454,A481,A482,A483,A484,A487,A512,A521,A525,A529	A267,A272,A275,A288,A289,A290,A305,A308,A309,A312,A317,A318,A332,A333,A334,A335,A336,A337,A338,A339,A340,A341,A373,A378,A379,A380,A381,A389,A398,A399,A400,A401
**Phospho-2-dehydro-3-deoxyheptonate aldolase AroG**	3KGF_1	PHE	A9003	A91,A92,A171,A174,A175,A178,B3,B5,B6,B55,B173	A87,A126,A130,A134,A248,A280,A281,A282,A283,A284,A306,A307,A337,A366,A369,A409,A411,A441
**Phospho-2-dehydro-3-deoxyheptonate aldolase AroG**	3KGF_2	TRP	A9004	A107,A110,A111,A123,A192,A194,A237,A238,A240,A241	A87,A126,A130,A134,A248,A280,A281,A282,A283,A284,A306,A307,A337,A366,A369,A409,A411,A441
**Glutamate receptor 3**	3LSW	4MP	A801	A105,A106,A107,A108,A217,A218,A219	A61,A62,A74,A89,A90,A91,A96,A111,A137,A138,A140,A141,A142,A143,A144,A174,A191,A192,A193,A196,A220
**Glutamate receptor 3**	3LSX	PZI	A802	A248,A252	A61,A62,A74,A89,A90,A91,A96,A111,A137,A138,A140,A141,A142,A143,A144,A174,A191,A192,A193,A196,A220
**Serum albumin**	3LU6	IMX	A587	A209,A212,A213,A216,A232,A235,A324,A3	A387,A388,A391,A392,A395,A403,A407

				27,A328,A347,A351,A354	,A410,A411,A414,A430,A431,A433,A434,A435,A437,A438,A449,A450,A453,A457,A485,A488,A489
**Glutamate receptor 3**	3M3F	P99	A800	A92,A104,A105,A106,A107,A108,A217,A218,A219,A239,A242,A247	A61,A62,A74,A89,A90,A91,A96,A111,A137,A138,A140,A141,A142,A143,A144,A174,A191,A192,A193,A196,A220
**Glutamate dehydrogenase 1, mitochondrial**	3MW9	NAI	A604	A195,A205,A206,A387,A388,A391,A392,A393,B85,B86,B115,B116,B119,B120,B121,B488,B491	A111,A114,A126,A167,A168,A211,A349,A374,A377,A378
**Prephenate dehydratase**	3MWB	PHE	B311	A226,A227,A228,A229,A230,B205,B208,B209,B210,B211,B212,B213,B232,B242,B244	A52,A53,A54,A55,A56,A57,A79,A80,A81,A82,A83,A166,A167,A168,A169,A170
**Pyruvate kinase PKM**	3N25	PRO	A1200	A42,A43,A69,A105,A463,A465,A467,A468,A469,A470	A73,A75,A77,A113,A114,A118,A243,A270,A272,A291,A292,A293,A294,A295,A296,A297,A327,A328,A360,A362
**Mitogen-activated protein kinase 8**	3O2M	46A	A701	A178,A180,A197,A198,A199,A230,A231,A234,A253,A255,A256,A259,B184,B255	A32,A33,A40,A52,A53,A55,A86,A108,A109,A110,A111,A112,A113,A114,A158,A168
**Phospho-2-dehydro-3-deoxyheptonate aldolase**	3PG9	TYR	A339	A31,A33,A34,A35,A36,A38,F1,F2,F40,F41,F42,F43,F45,F65,F66	A102,A131,A132,A164,A186,A247,A272,A309
**UDP-glucose 6-dehydrogenase**	3PJG	UGA	A902	A256,A257,A259,A284,A319,A320,A322,A323,A324,A326,A345	A131,A161,A162,A163,A164,A165,A220,A275,A276,A280
**UDP-glucose 6-dehydrogenase**	3PTZ	UDX	A501	A131,A161,A162,A163,A164,A165,A220,A224,A227,A231,A265,A266,A267,A269,A272,A273,A276,A277,A338,A339,A442,B260	A131,A161,A162,A163,A164,A165,A220,A275,A276,A280
**Cyclin-dependent kinase 2**	3PXF	2AN	A304,A305	A15,A33,A35,A37,A52,A55,A56,A64,A66,A69,A71,A76,A78,A80,A144,A145,A146,A154	A10,A11,A12,A13,A14,A15,A16,A17,A18,A31,A33,A64,A80,A81,A82,A83,A84,

					A85,A86,A89,A127,A129,A130,A131,A132,A133,A134,A144,A145,A162
**Tyrosine-protein kinase ABL1**	3PYY	3YY	A538	A356,A359,A360,A363,A448,A451,A452,A481,A482,A483,A484,A487	A267,A272,A275,A288,A289,A290,A305,A308,A309,A312,A317,A318,A332,A333,A334,A335,A336,A337,A338,A339,A340,A341,A373,A378,A379,A380,A381,A389,A398,A399,A400,A401
**Prostaglandin G/H synthase 2**	3QH0	PLM	A625	A116,A120,A205,A348,A349,A353,A355,A385,A387,A523,A526,A527,A530,A531	B75,B102,B106,B191,B330,B334,B335,B338,B339,B341,B345,B367,B370,B371,B373,B504,B508,B509,B510,B511,B512,B513,B514,B516,B517,B520
**Ribonucleoside-diphosphate reductase 1 subunit alpha**	3R1R	ATP	A762	A9,A15,A16,A17,A18,A21,A22,A25,A55,A59,A91	A155,A207,A208,A209,A210,A224,A225,A226,A251,A252,A253,A254,A301,A437,A438,A439,A441,A442,A464,A620,A621,A622,A623,A624,A625,A626,A694
**Glutaminase kidney isoform, mitochondrial**	3UO9	04A	B2	A320,A321,A322,A323,A324,A325,A327,A394,B320,B321,B322,B323,B324,B325,B394,D317	A249,A284,A285,A286,A287,A289,A335,A381,A387,A388,A414,A415,A418,A466,A482,A483,A484,A485
**Kinesin-like protein KIF11**	3ZCW	4A2	A1366	A104,A266,A269,A270,A287,A288,A289,A292,A293,A295,A296,A297,A299,A300,A332,A352,A353,A355,A356	A24,A25,A26,A27,A74,A76,A78,A105,A106,A107,A108,A109,A110,A111,A112,A113,A114,A118,A132,A232,A233,A265,A335
**Glutamate racemase**	4B1F	KRH	A1256	A10,A11,A13,A17,A149,A150,A152,A154,A182,A183,A186,A248,A252,A253	A7,A8,A9,A11,A12,A37,A38,A39,A40,A41,A69,A70,A71,A72,A73,A116,A119,A146,A150,A180,A181,A182,A183
**Pyruvate kinase PKM**	4B2D	SER	A1532	A43,A44,A45,A46,A70,A106,A464,A468,A4	A73,A75,A77,A113,A114,A118,A243,A2

				69,A470,A471	70,A272,A291,A292,A293,A294,A295,A296,A297,A327,A328,A360,A362
**Kinesin-like protein KIF11**	4BBG	V02	A1370	A112,A116,A117,A118,A119,A130,A132,A133,A137,A211,A214,A215,A218,A221	A24,A25,A26,A27,A74,A76,A78,A105,A106,A107,A108,A109,A110,A111,A112,A113,A114,A118,A132,A232,A233,A265,A335
**5'-AMP-activated protein kinase catalytic subunit alpha-2**	4CFE	992	A1553	A11,A18,A24,A28,A29,A31,A46,A48,A88,A90,B81,B83,B106,B107,B111,B113	A22,A23,A24,A25,A26,A30,A43,A45,A64,A77,A91,A93,A94,A95,A96,A97,A98,A99,A100,A141,A143,A144,A145,A146,A156,A157,A158
**Mitogen-activated protein kinase 14**	4E6C	0O8	A500	A195,A196,A197,A255	A30,A33,A38,A39,A40,A51,A52,A53,A71,A75,A84,A85,A86,A88,A104,A105,A106,A107,A108,A109,A110,A111,A112,A115,A154,A156,A157,A158,A167,A168
**Pyruvate kinase PKM**	4G1N	NZT	A603	A26,A30,A353,A354,A389,A390,A393,A394,A397,B26,B27,B30,B311,B353,B354,B390,B394	A73,A75,A77,A113,A114,A118,A243,A270,A272,A291,A292,A293,A294,A295,A296,A297,A327,A328,A360,A362
**2-dehydro-3-deoxyphosphoheptonate aldolase**	4GRS	TYR	A401	A1,A40,A41,A42,A43,A45,A66,C31,C33,C34,C35,C36,C38	A102,A131,A132,A164,A186,A247,A272,A309
**Glucose-1-phosphate thymidylyltransferase**	4HO6	UTP	A301	A43,A112,A113,A114,A115,A116,A117,A248,A249,A253,A254,A257	A8,A9,A10,A11,A12,A13,A14,A15,A16,A25,A26,A54,A55,A82,A84,A85,A86,A87,A88,A90,A108,A109,A110,A111,A162,A194,A196,A225,A227
**Pyruvate kinase 1**	4HYW	FDP	A503	A400,A401,A402,A403,A405,A406,A454,A457,A481,A482,A486,A487,A488,A489,A490	A50,A52,A54,A84,A85,A89,A212,A213,A239,A241,A260,A261,A262,A263,A264

					,A265,A266,A296,A297,A329,A331
**Isocitrate dehydrogenase [NADP], mitochondrial**	4JA8	1K9	A502	A164,A294,A297,A298,A306,A311,A312,A315,A316,A319,A320,B160,B164,B294,B297,B298,B306,B311,B312,B315,B316,B319,B320	A57,A59,A62,A112,A113,A114,A115,A116,A117,A118,A122,A136,A326,A327,A328,A345,A346,A347,A348,A349,A350,A351,A352,A353,A354,A365,A366,A367,A368,A412,A414,A422
**Glutaminase kidney isoform, mitochondrial**	4JKT	04A	D701	A322,A325,A326,A327,A328,A329,A330,A399,C322,D322,D325,D326,D327,D328,D329,D330,D399	A249,A284,A285,A286,A287,A289,A335,A381,A387,A388,A414,A415,A418,A466,A482,A483,A484,A485
**N-acetylglutamate kinase / N-acetylglutamate synthase**	4KZT	ARG	A501	A28,A206,A225,A265,A277,A278,A280,A281,A282,A283,A285,A286,A287,A365	A307,A312,A354,A355,A356,A357,A358,A359,A363,A364,A365,A366,A367,A368,A369,A370,A371,A391,A392,A394,A395,A396,A397,A398,A399,A401,A402
**HD domain protein**	4LRL	TTP	B503	A55,A56,A197,A241,A245,B15,B16,B17,D206,D209,D223	B48,B49,B50,B51,B52,B63,B66,B111,B114,B118,B119,B122,B129,B183,B184,B187,B191,B235,B239,B242,B243,B244,B248,B252,B368,B369
**Glycogen phosphorylase, muscle form**	4MRA	QUE	A901	A121,A124,A495,A544,A545,A548,A551,A552,A655	A134,A135,A675,A676
**ATP-dependent 6-phosphofructokinase**	4PFK	ADP	A326	A154,A185,A187,A211,A212,A213,A214,A215	A9,A10,A11,A12,A41,A71,A72,A73,A74,A75,A76,A77,A82,A101,A102,A103,A104,A105,A106,A107,A108,A109,A111,A124,A125,A129,A171

Supplementary Table 2: **Allosteric site quantile scores of proteins in Supplementary Table 1.** The results from four statistical scores described in Amor *et al.*. Average site residue and bond quantile scores are compared with those of 1000 surrogate sites of the same size. The difference is shown in bold if it is above 0, and starred if it is above the 95% confidence interval. The proportion of bonds with $p_{b,\text{allo}} > 0.95$ and the average reference quantile score $\overline{p^{\text{ref}}_{b,\text{allo}}}$ are shown in bold if they are above the expected values of 0.05 and 0.5 respectively.

Protein	PDB	$\overline{p_{R,\text{allo}}} - \langle\overline{p_{R,\text{site}}}\rangle_{\text{surr}}$	$\overline{p_{b,\text{allo}}} - \langle\overline{p_{b,\text{site}}}\rangle_{\text{surr}}$	$P(p_{b,\text{allo}} > 0.95)$	$\overline{p^{\text{ref}}_{b,\text{allo}}}$	Summary
**Fructose-1,6-bisphosphatase class 1**	2Q8M_2	**0.38***	**0.2***	**0.19**	**0.54**	●●●●
**Cytochrome P450 3A4**	1W0F	**0.37***	**0.059***	0.0078	**0.5**	●●○●
**Tryptophan 2,3-dioxygenase**	2NW8	**0.34***	**0.1***	**0.13**	**0.58**	●●●●
**Parathion hydrolase**	1QW7	**0.32***	-0.14	0.022	**0.51**	●○○●
**Glutamate dehydrogenase 1, mitochondrial**	3ETG	**0.31***	**0.17***	**0.085**	**0.75**	●●●●
**Lysine-sensitive aspartokinase 3**	2J0X	**0.3***	**0.19***	**0.11**	**0.62**	●●●●
**Aspartate carbamoyltransferase regulatory chain**	2BE9	**0.29***	**0.25***	**0.1**	0.11	●●●○
**Copper-containing nitrite reductase**	1ZDS	**0.28***	**0.13***	**0.12**	**0.78**	●●●●
**Androgen receptor**	2QPY	**0.26***	**0.22***	**0.11**	**0.53**	●●●●
**Tyrosine-protein kinase ABL1**	3K5V	**0.26***	**0.15***	0.034	**0.63**	●●○●
**Fructose-1,6-bisphosphatase isozyme 2**	3IFA	**0.26***	**0.11***	**0.16**	**0.61**	●●●●
**Hemoglobin subunit beta**	1B86	**0.26***	-0.0016	**0.24**	**0.54**	●○●●
**Tyrosine-protein kinase ABL1**	3PYY	**0.22***	**0.11***	0.033	**0.59**	●●○●

**Glutamate receptor 3**	3LSX	**0.22***	**0.022***	**0.2**	0.41	●●●○
**Isocitrate dehydrogenase kinase/phosphatase**	3EPS	**0.2***	**0.17***	0.047	**0.76**	●●○●
**Serum albumin**	3LU6	**0.2***	**0.13***	**0.072**	**0.66**	●●●●
**HD domain protein**	3IRH	**0.2***	**0.04***	**0.099**	**0.66**	●●●●
**Indole-3-pyruvate decarboxylase**	2Q5O	**0.19***	**0.057***	**0.12**	**0.7**	●●●●
**Toxin A**	3HO6	**0.19***	**0.028***	**0.13**	**0.6**	●●●●
**Pyruvate kinase PKM**	4G1N	**0.19***	**0.019***	0.029	**0.71**	●●○●
**Anthranilate synthase component 1**	1I7S	**0.18***	**0.1***	0.04	**0.61**	●●○●
**Glycogen phosphorylase, muscle form**	1Z8D_1	**0.18***	-0.064	0.038	0.41	●○○○
**Glycogen phosphorylase, liver form**	1EM6	**0.17***	**0.093***	**0.062**	**0.73**	●●●●
**Pyruvate dehydrogenase kinase isozyme 2**	2BU8	**0.17***	**0.081***	**0.065**	**0.65**	●●●●
**Pyruvate kinase PKM**	3N25	**0.17***	**0.077***	**0.091**	**0.73**	●●●●
**Fructose-1,6-bisphosphatase class 1**	2Q8M_1	**0.17***	-0.035	0.015	0.48	●○○○
**Casein kinase II subunit alpha**	3H30	**0.16***	**0.1***	**0.086**	**0.75**	●●●●
**ATP-dependent 6-phosphofructokinase**	4PFK	**0.16***	**0.098***	**0.12**	0.41	●●●○
**ATP phosphoribosyltransferase**	1NH8	**0.16***	**0.096***	**0.065**	0.087	●●●○
**Carbamoyl-phosphate**	1CE8_1	**0.16***	**0.041***	**0.12**	**0.65**	●●●●

**synthase large chain**						
**Glutamate dehydrogenase 1, mitochondrial**	3ETE_1	**0.15***	**0.082***	0.038	**0.73**	●●○●
**Ribonucleoside-diphosphate reductase 1 subunit alpha**	2R1R	**0.15***	**0.062***	**0.13**	**0.63**	●●●●
**Uridylate kinase**	2V4Y	**0.14***	**0.059***	**0.11**	**0.66**	●●●●
**Glutamate racemase**	3HFR	**0.14***	**0.055***	0.038	**0.62**	●●○●
**Seminal ribonuclease**	11BG	**0.14***	**0.042***	**0.081**	**0.51**	●●●●
**Tyrosine-protein phosphatase non-receptor type 1**	1T49	**0.14***	**0.029***	0.045	0.49	●●○○
**Serum albumin**	2BXA	**0.14***	**0.024***	**0.062**	**0.51**	●●●●
**Uridylate kinase**	3EK5	**0.14***	**0.011***	**0.11**	**0.62**	●●●●
**Glycogen phosphorylase, liver form**	3CEH	**0.14***	**0.0012**	**0.078**	**0.67**	●○●●
**Acetyl-CoA carboxylase**	1W96	**0.14***	-0.015	**0.057**	**0.66**	●○●●
**Leukotriene A-4 hydrolase**	3FUD	**0.14***	-0.052	**0.067**	0.41	●○●○
**Glucose-1-phosphate thymidylyltransferase 1**	1H5S	**0.13***	**0.11***	**0.098**	**0.72**	●●●●
**Androgen receptor**	2YLO	**0.13***	**0.09***	0.014	0.47	●●○○
**5'-AMP-activated protein kinase catalytic subunit alpha-2**	4CFE	**0.12***	**0.16***	**0.069**	**0.73**	●●●●
**Phospho-2-dehydro-3-deoxyheptonate aldolase**	3PG9	**0.12***	**0.09***	**0.11**	**0.72**	●●●●
**Androgen receptor**	2YHD	**0.12***	**0.078***	0.037	**0.56**	●●○●
**Pyruvate kinase PKM**	4B2D	**0.11***	**0.11***	**0.13**	**0.74**	●●●●

**Glucosamine-6-phosphate isomerase 1**	1NE7	**0.11***	**0.07***	**0.1**	**0.66**	●●●●
**Fructose-1,6-bisphosphatase 1**	1FRP	**0.11***	**0.033***	**0.067**	**0.54**	●●●●
**Uracil phosphoribosyltransferase**	1JLR	**0.1***	**0.017***	**0.082**	**0.65**	●●●●
**Glucose-1-phosphate thymidylyltransferase**	1LVW	**0.1***	-0.021	**0.07**	**0.6**	●○●●
**Carbamoyl-phosphate synthase large chain**	1CE8_2	**0.099***	**0.068***	**0.15**	**0.68**	●●●●
**Hemoglobin subunit beta**	2D60	**0.093***	**0.062***	0.034	**0.66**	●●○●
**L-asparaginase 1**	2HIM	**0.08***	**0.15***	**0.097**	**0.76**	●●●●
**Response regulator PleD**	1W25	**0.073***	**0.092***	**0.14**	**0.73**	●●●●
**Putative deoxycytidylate deaminase**	2HVW	**0.067***	-0.15	**0.13**	0.39	●○●○
**Prephenate dehydratase**	2QMX	**0.065***	-0.0045	0.042	**0.65**	●○○●
**Glycogen phosphorylase, muscle form**	3E3N	**0.06***	-0.086	0.02	**0.57**	●○○●
**Mitogen-activated protein kinase 8**	3O2M	**0.059***	-0.0006	0.0047	**0.57**	●○○●
**Isocitrate dehydrogenase [NADP], mitochondrial**	4JA8	**0.053***	-0.074	0.0028	0.48	●○○○
**Ribose-phosphate pyrophosphokinase**	1DKU	**0.05***	**0.039***	**0.17**	**0.62**	●●●●
**Phospho-2-dehydro-3-deoxyheptonate aldolase AroG**	3KGF_2	**0.037***	**0.018***	0.046	**0.57**	●●○●
**Chorismate mutase**	3CSM	**0.037***	**0.0082***	0.012	0.46	●●○○

**Cytosolic purine 5'-nucleotidase**	2XJC	**0.031***	**0.058***	**0.1**	**0.58**	●●●●
**Glucose-1-phosphate thymidylyltransferase**	1G3L	**0.021***	**0.056***	**0.071**	**0.71**	●●●●
**Glutamate receptor 3**	3M3F	**0.019***	**0.038***	0.045	**0.51**	●●○●
**Probable aspartokinase**	3C1N	**0.017***	-0.11	0	0.47	●○○○
**Mitogen-activated protein kinase 14**	4E6C	**0.015***	-0.018	**0.11**	0.24	●○●○
**ATP-dependent 6-phosphofructokinase isozyme 1**	1PFK	**0.014***	-0.09	**0.059**	0.46	●○●○
**Anaerobic ribonucleoside-triphosphate reductase**	1H78	**0.01***	-0.00072	**0.053**	0.45	●○●○
**Multifunctional 2-oxoglutarate metabolism enzyme**	2Y0P	**0.0075***	**0.012***	0.046	**0.59**	●●○●
**Kinesin-like protein KIF11**	4BBG	**0.0056***	-0.04	0.047	**0.53**	●○○●
**UDP-glucose 6-dehydrogenase**	3PTZ	**0.0052***	-0.16	**0.063**	0.43	●○●○
**HD domain protein**	4LRL	**0.0029***	-0.012	**0.067**	**0.64**	●○●●
**Glycogen phosphorylase, muscle form**	2IEG	-0.0011	-0.0095	0.034	**0.68**	○○○●
**Glucose-1-phosphate thymidylyltransferase**	1MP3	-0.0076	**0.11***	**0.11**	**0.67**	○●●●
**Cyclin-dependent kinase 2**	3PXF	-0.0093	-0.073	0.032	0.46	○○○○
**Glucose-1-phosphate thymidylyltransferase**	4HO6	-0.0098	**0.0074***	0.048	**0.54**	○●○●

**Myosin-2 heavy chain**	2JHR	-0.011	**0.053***	**0.067**	**0.59**	○●●●
**Pyruvate kinase 1**	4HYW	-0.015	-0.0059	0.035	0.43	○○○○
**N-acetylglutamate kinase / N-acetylglutamate synthase**	4KZT	-0.019	**0.049***	**0.053**	**0.61**	○●●●
**Myosin-2 heavy chain**	3BZ7	-0.021	-0.031	**0.061**	**0.64**	○○●●
**Glutamate racemase**	2VVT	-0.027	**0.051***	**0.066**	**0.59**	○●●●
**Glutamine--fructose-6-phosphate aminotransferase**	2PUV	-0.028	**0.11***	**0.064**	**0.7**	○●●●
**Phospho-2-dehydro-3-deoxyheptonate aldolase AroG**	3KGF_1	-0.029	-0.059	0.0078	**0.53**	○○○●
**Prostaglandin G/H synthase 2**	3QH0	-0.03	-0.095	0.0047	0.45	○○○○
**Hemoglobin subunit alpha**	1IWH	-0.032	-0.13	0.034	0.47	○○○○
**Kinesin-like protein KIF11**	3ZCW	-0.034	**0.078***	0.035	**0.62**	○●○●
**NAD(P)-dependent glyceraldehyde-3-phosphate dehydrogenase**	1UXV	-0.034	-0.074	0.027	0.44	○○○○
**Pyruvate kinase 1**	1A3W	-0.037	**0.058***	**0.07**	0.49	○●●○
**Phospho-2-dehydro-3-deoxyheptonate aldolase, tyrosine-inhibited**	1OF6	-0.037	-0.034	0.04	**0.54**	○○○●
**Phospho-2-dehydro-3-deoxyheptonate aldolase, Phe-sensitive**	1KFL	-0.038	-0.049	0.041	**0.59**	○○○●
**Ornithine decarboxylase**	1NJJ	-0.04	**0.085***	**0.059**	**0.64**	○●●●
**Glutamate racemase**	4B1F	-0.04	-0.02	0.014	**0.59**	○○○●
**Glutamate dehydrogenase 1,**	3MW9	-0.041	**0.017***	0.045	**0.64**	○●○●

**mitochondrial**						
**NAD-dependent malic enzyme, mitochondrial**	1GZ3	-0.045	-0.051	0.038	**0.65**	○○○●
**Glycogen phosphorylase, muscle form**	4MRA	-0.047	-0.15	0.035	0.49	○○○○
**Glutamate receptor 3**	3LSW	-0.049	-0.047	**0.058**	0.47	○○●○
**Glutaminase kidney isoform, mitochondrial**	4JKT	-0.054	-0.064	0.0097	**0.56**	○○○●
**Acetylglutamate kinase, chloroplastic**	2RD5	-0.073	-0.058	0.01	**0.55**	○○○●
**Glutamate racemase**	2W4I	-0.087	-0.045	0.04	**0.56**	○○○●
**Prephenate dehydratase**	3MWB	-0.095	-0.11	0.014	**0.5**	○○○●
**Glutamate dehydrogenase 1, mitochondrial**	3ETE_2	-0.11	-0.088	0.047	**0.54**	○○○●
**Pyruvate kinase PKLR**	2VGI	-0.13	-0.028	0.018	**0.65**	○○○●
**Glutaminase kidney isoform, mitochondrial**	3UO9	-0.16	-0.084	0.011	**0.6**	○○○●
**UDP-glucose 6-dehydrogenase**	3PJG	-0.16	-0.1	0.043	0.42	○○○○
**2-dehydro-3-deoxyphosphoheptonate aldolase**	4GRS	-0.16	-0.13	0.011	**0.53**	○○○●
**Pyruvate kinase PKM**	3H6O	-0.18	-0.12	0.045	**0.53**	○○○●
**Pyruvate kinase**	3HQP	-0.19	-0.2	0.02	0.45	○○○○
**Ribonucleoside-diphosphate reductase 1 subunit alpha**	3R1R	-0.2	-0.18	0	0.39	○○○○
**Glycogen phosphorylase,**	3BCR	-0.23	-0.054	0.029	**0.67**	○○○●

**muscle form**						
**Amino-acid acetyltransferase**	3D2P	-0.27	-0.18	0.0075	0.32	○○○○
**D-3-phosphoglycerate dehydrogenase**	2PA3	-0.29	-0.16	0	0.082	○○○○
**Sulfate adenylyltransferase**	1M8P	-0.34	-0.3	0	0.24	○○○○
**ATP phosphoribosyltransferase**	2VD3	-0.4	-0.26	0	0.29	○○○○
**D-3-phosphoglycerate dehydrogenase**	3DC2	-0.42	-0.4	0	0.02	○○○○
**Glycogen phosphorylase, muscle form**	1Z8D_2	-0.43	-0.26	0	0.45	○○○○
**4-hydroxy-tetrahydrodipicolinate synthase**	2ATS	-0.44	-0.19	0	0.29	○○○○
